# Suspected Secondary Intraocular Lymphoma With Choroidal Involvement Following Testicular Diffuse Large B-cell Lymphoma: A Case Report

**DOI:** 10.7759/cureus.106563

**Published:** 2026-04-07

**Authors:** Battuya Ganbold, Bayasgalan Purevdorj, Quan Dong Nguyen, Dariimaa Ganbat

**Affiliations:** 1 Department of Ophthalmology, Bolor Melmii Hospital, Ulaanbaatar, MNG; 2 Center of Academic Education, Graduate School of Medical Science, Mongolian National University of Medical Sciences, Ulaanbaatar, MNG; 3 Department of Ophthalmology, Mongolian National University of Medical Sciences, Ulaanbaatar, MNG; 4 Spencer Center for Vision Research, Byers Eye Institute at Stanford University, Palo Alto, USA; 5 Center of International Cyber Education, Graduate School of Medical Science, Mongolian National University of Medical Sciences, Ulaanbaatar, MNG

**Keywords:** choroidal lymphoma, multimodal imaging, secondary intraocular lymphoma, testicular diffuse large b-cell lymphoma, vitreoretinal lymphoma

## Abstract

Secondary intraocular lymphoma is an uncommon manifestation of systemic lymphoma and may relapse in immune-privileged sites years after apparent remission. We report the case of a 67-year-old man with a history of right testicular diffuse large B-cell lymphoma (DLBCL) treated with orchiectomy and systemic chemotherapy who presented five years later with painless progressive vision loss in the left eye. Examination revealed visual acuity of 20/80, fibrinoid material with hemorrhage in the anterior chamber, and yellow-white choroidal infiltrates extending from the optic disc toward the periphery with associated subretinal fluid. Multimodal imaging demonstrated subretinal infiltrates and diffuse choroidal thickening. Diagnostic pars plana vitrectomy was performed during repair of an associated retinal detachment. Routine cytology was nondiagnostic, MYD88 L265P testing was negative, and flow cytometry using a limited panel identified an abnormal lambda-restricted B-cell population comprising 45.2% of CD45+ events, without definitive morphologic confirmation. In the context of the patient’s prior lymphoma history, supportive multimodal ocular findings, and exclusion of major infectious and inflammatory etiologies, the overall clinicopathologic impression favored secondary intraocular lymphoma with choroidal involvement. Systemic restaging showed no active extraocular or central nervous system disease. Treatment with oral ibrutinib 560 mg daily was associated with clinical stability over 24 months, improvement of visual acuity to 20/40, resolution of subretinal fluid, stable residual choroidal changes, and no evidence of systemic recurrence. This case highlights the importance of long-term ophthalmic surveillance in patients with testicular DLBCL and the integration of multimodal imaging, ocular fluid analysis, and systemic restaging in the evaluation of suspected ocular relapse.

## Introduction

Intraocular lymphoma is a rare but clinically significant malignancy that may present either as primary ocular disease or as secondary involvement from systemic lymphoma [1,2]. Secondary intraocular lymphoma most commonly affects the choroid and is an important masquerade syndrome because it may closely resemble inflammatory or infectious chorioretinal disorders in routine practice. Diagnosis is often challenging because the clinical findings can overlap with uveitic disease, while malignant cells may be sparse or fragile in ocular fluid specimens, limiting the sensitivity of cytologic confirmation [1-3].

Among systemic lymphomas, testicular diffuse large B-cell lymphoma (DLBCL) is particularly notable for relapse in immune-privileged sites, including the contralateral testis, central nervous system, and eye. Immune-privileged sites are anatomical compartments in which local immune responses are relatively restricted, potentially allowing malignant cells to evade immune surveillance and persist despite apparent systemic remission [3-5]. Ocular relapse after testicular DLBCL may occur months or years after apparently successful treatment, underscoring the importance of prolonged surveillance in this high-risk population [4,5].

Because of its rarity, delayed presentation, and overlap with inflammatory ocular disease, suspected secondary intraocular lymphoma requires a high index of suspicion, careful multimodal imaging, ocular fluid or tissue analysis when feasible, and systemic restaging [6-9]. We report a patient with unilateral choroidal-predominant intraocular relapse and anterior segment abnormalities occurring five years after treatment of testicular DLBCL, highlighting the diagnostic challenges of this presentation and the durable clinical control achieved with oral ibrutinib.

## Case presentation

A 67-year-old man presented in December 2021 with gradual, painless progressive visual loss in the left eye over the preceding four months; exact visual acuity at symptom onset was not documented in the available records. He denied ocular pain, redness, photophobia, floaters, or flashes and reported no fever, night sweats, weight loss, or neurologic symptoms.

His past medical history was notable for right testicular DLBCL diagnosed in March 2017. He underwent right orchiectomy, and pathology reportedly showed CD3-negative, CD5-positive, CD20-positive, BCL1-negative, BCL2-positive, and ALK1-negative lymphoma. Fluorescence in situ hybridization had not been performed, and the exact systemic chemotherapy regimen could not be confirmed from the accessible records. He achieved complete remission and remained under oncologic surveillance without documented recurrence until the current ocular presentation.

**Figure 1:**
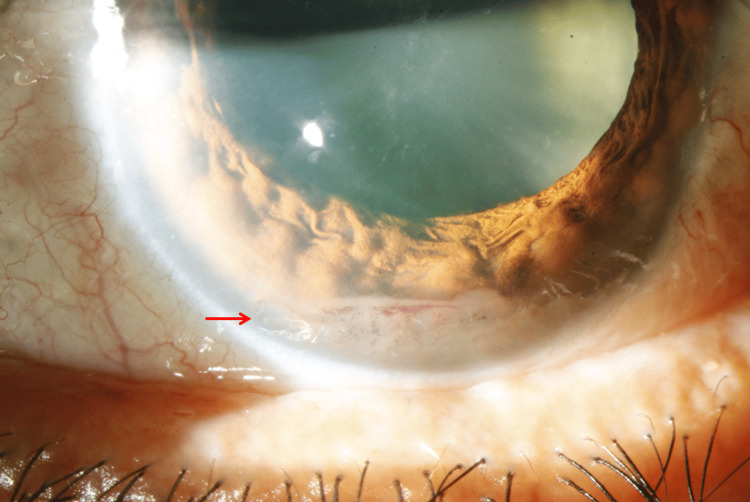
Slit-lamp photograph of the left eye at presentation Slit-lamp photograph of the left eye at initial presentation demonstrating inferior anterior chamber fibrinoid material with associated hemorrhagic debris (red arrow). No keratic precipitates or synechiae were identified clinically. These anterior-segment findings were present in the setting of unilateral painless progressive visual loss and raised concern for an ocular masquerade syndrome.

**Figure 2:**
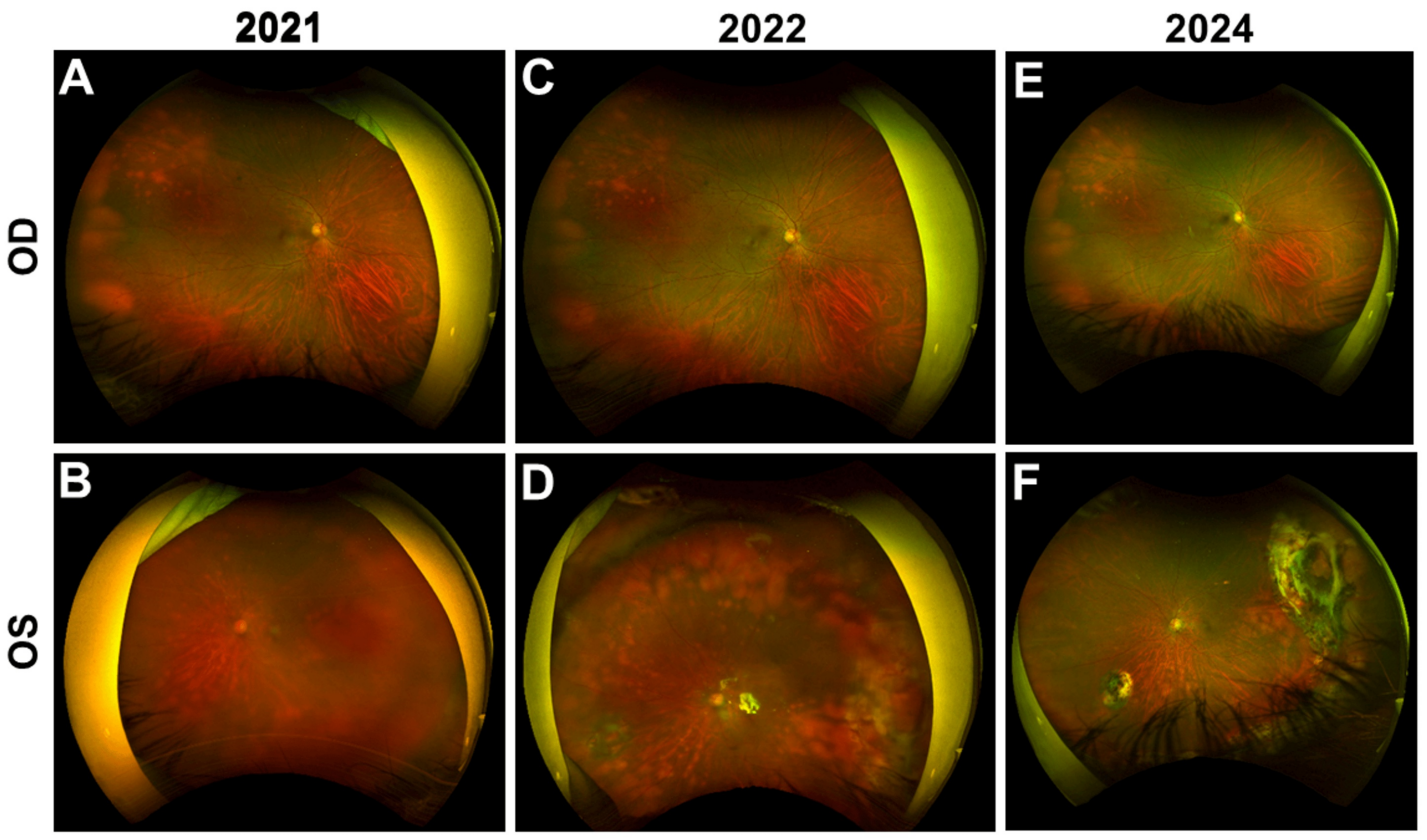
Serial ultra-widefield fundus photographs of both eyes obtained in 2021, 2022, and 2024 A, C, E (OD): right eye remained without clinically significant posterior segment abnormality. B, D, F (OS): left eye demonstrated yellow-white choroidal infiltrative lesions extending from the peripapillary region toward the peripheral retina, with lesions becoming more conspicuous on follow-up imaging and later showing partial regression/stability with residual pigmentary change after treatment. This serial presentation illustrates the longitudinal evolution of the choroidal-predominant process. OD, oculus dexter (right eye); OS, oculus sinister (left eye)

At initial ophthalmic examination, best-corrected visual acuity was 20/30 in the right eye and 20/80 in the left eye. Intraocular pressure was 13 mmHg in the right eye and 20 mmHg in the left eye. The right eye was unremarkable. Slit-lamp examination of the left eye demonstrated fibrinoid clumps with associated hemorrhage in the inferior anterior chamber without keratic precipitates, significant anterior chamber cell reaction, or synechiae (Figure 1). There was no clinically significant vitritis at presentation. Fundus examination of the left eye showed yellowish-white choroidal infiltrates extending from the optic disc toward the peripheral retina with overlying subretinal fluid and mild optic disc swelling (Figures 2A, 2B).

**Figure 3:**
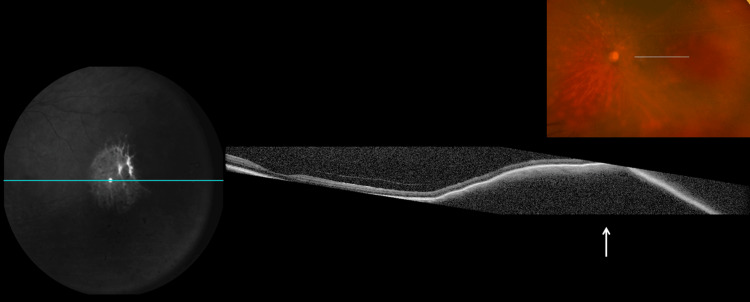
Optical coherence tomography of the left eye Optical coherence tomography of the left eye demonstrating hyperreflective subretinal infiltrative material, associated subretinal fluid (arrow), irregular retinal pigment epithelium elevation/undulation, and diffuse choroidal thickening. The inset fundus image shows the scan location through the involved posterior pole. These layer-specific changes supported choroidal involvement by a lymphoma-related infiltrative process.

**Figure 4:**
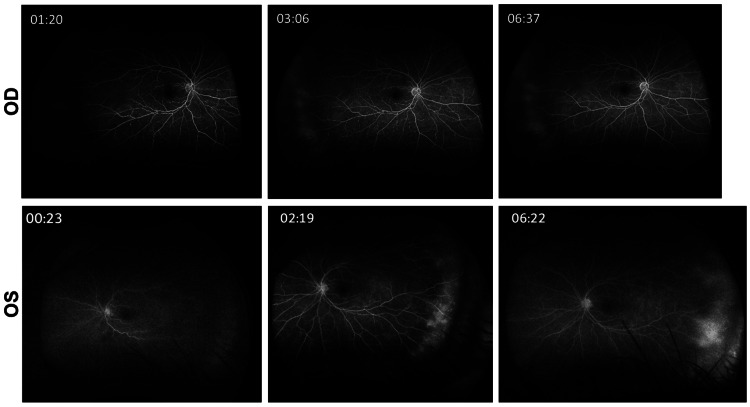
Fluorescein angiography of both eyes (2021 visit) Fluorescein angiography obtained in the early, mid, and late phases. Top row (OD): no major angiographic abnormality; Bottom row (OS): hypofluorescent areas corresponding to the choroidal infiltrates with progressive peripheral vascular leakage and late staining. These findings complemented the fundus and optical coherence tomography abnormalities and supported an infiltrative rather than purely inflammatory posterior segment process. OD, oculus dexter (right eye); OS, oculus sinister (left eye)

**Figure 5:**
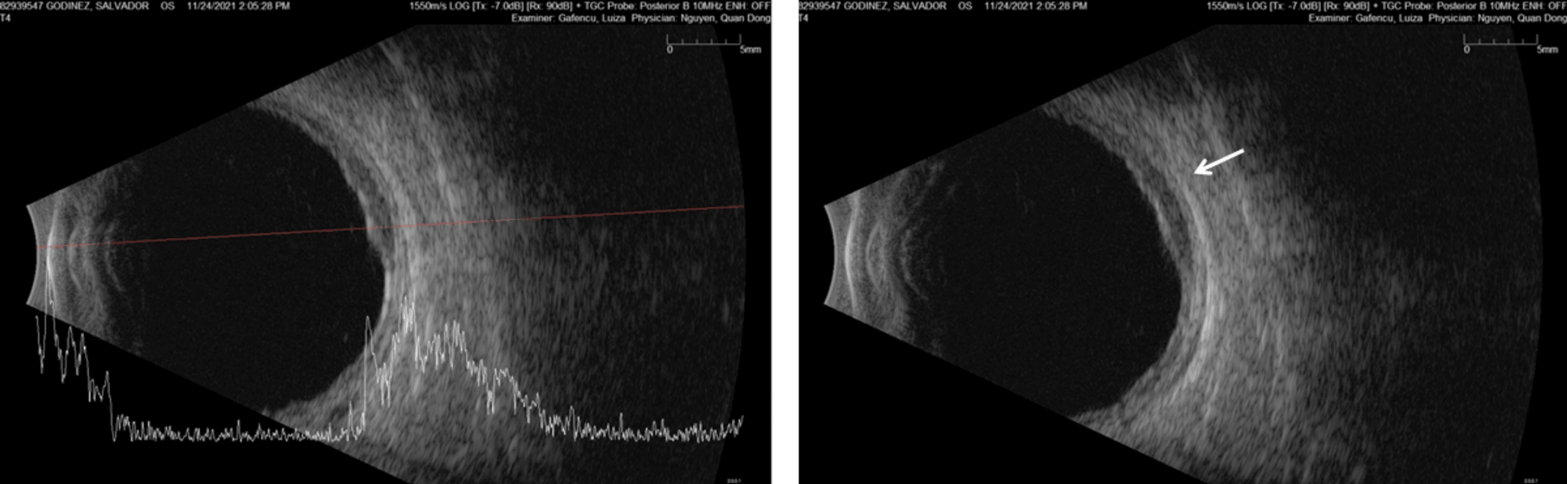
B-scan ultrasonography of the left eye (2021 visit) B-scan ultrasonography of the left eye demonstrating diffuse choroidal thickening measuring approximately 2.8–3.2 mm with medium-to-high internal reflectivity. No overt scleral involvement, extrascleral extension, or vitreous opacities were identified at presentation. The quantitative thickness measurement improves comparison with similar reported cases of choroidal lymphoma. OD, oculus dexter (right eye); OS, oculus sinister (left eye)

Optical coherence tomography of the left eye demonstrated subretinal infiltrates with associated subretinal fluid, irregular retinal pigment epithelium elevation, and diffuse choroidal thickening (Figure 3). Fluorescein angiography showed peripheral vascular leakage with hypofluorescent areas corresponding to the infiltrates and late staining (Figure 4). B-scan ultrasonography demonstrated diffuse choroidal thickening measuring approximately 2.8-3.2 mm with high internal reflectivity and no vitreous opacities at presentation (Figure 5). Indocyanine green angiography performed later during follow-up demonstrated multiple hypocyanescent spots corresponding to choroidal lesions (not shown).

Laboratory evaluation showed no major abnormalities on complete blood count, comprehensive metabolic panel, lactate dehydrogenase, or urinalysis. HIV serology, syphilis serology, toxoplasma serology, and herpes simplex virus polymerase chain reaction were negative. HLA-B27 and HLA-B51 were negative, whereas HLA-A29 was positive. Although the absence of significant vitritis and the positive HLA-A29 result initially broadened the inflammatory differential diagnosis, isolated HLA-A29 positivity was not considered diagnostic; in the setting of unilateral disease, prior testicular DLBCL, choroidal infiltrative lesions with subretinal fluid, diffuse choroidal thickening, and vitreous evidence of a monoclonal B-cell population, the overall picture remained most consistent with secondary intraocular lymphoma. Ancillary molecular, immunogenetic, and systemic imaging findings relevant to diagnostic evaluation and staging are summarized in Appendix A.

On December 16, 2021, the patient underwent diagnostic pars plana vitrectomy with vitreous biopsy. An associated retinal detachment was identified intraoperatively and repaired with drainage retinotomy, scleral buckle, endolaser photocoagulation, and silicone oil injection. The patient had not presented with classic acute retinal detachment symptoms, and the available records did not permit reliable retrospective classification of the detachment by type, extent, or macular status. Microbiologic cultures were negative. MYD88 L265P testing was negative. Routine cytology showed retinal pigment epithelial cells and inflammatory cells without definitive malignant cells. Flow cytometry, performed using a limited immunophenotypic panel, identified an abnormal lambda-restricted B-cell population comprising 45.2% of CD45+ events, with expression of CD19, CD20, and CD22 and absence of CD5 and CD10; sample cellularity was low (cell count, 0.07 K/μL), although detailed gating plots were not available for review. Although the result was not independently diagnostic of recurrent DLBCL, the combined ocular phenotype, prior lymphoma history, exclusion of major infectious etiologies, and monoclonal vitreous findings favored secondary intraocular lymphoma with choroidal involvement. Detailed vitreous cytology and flow cytometry findings from ocular specimens are provided in Appendix B.

The patient began oral ibrutinib 560 mg once daily on February 24, 2022 (Figures 2C, 2D). After multidisciplinary discussion, ibrutinib was selected because systemic restaging revealed no active extraocular or central nervous system disease, and the treating team preferred a less toxic systemic option over more intensive methotrexate-based therapy in this older patient. Intravitreal chemotherapy and radiotherapy were deferred on the same basis. The treatment was well tolerated, with serial clinical and laboratory monitoring showing no bleeding, infection, atrial fibrillation, clinically significant cytopenia, or other treatment-limiting toxicity. Systemic restaging with chest computed tomography and positron emission tomography/computed tomography in February 2022 showed no active systemic lymphoma. Brain magnetic resonance imaging obtained during surveillance in November 2022 showed no central nervous system involvement. 

**Figure 6:**
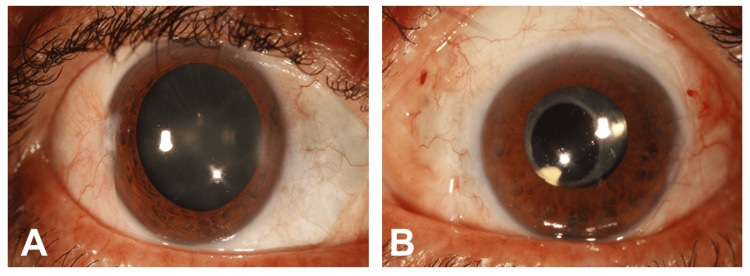
Slit-lamp photographs of the right (A) and left (B) eyes at follow-up Anterior chamber fibrinoid material has resolved, with a clear interval improvement in the inflammatory appearance. This objective interval change is consistent with the clinical improvement observed during 2024-visit.

**Figure 7:**
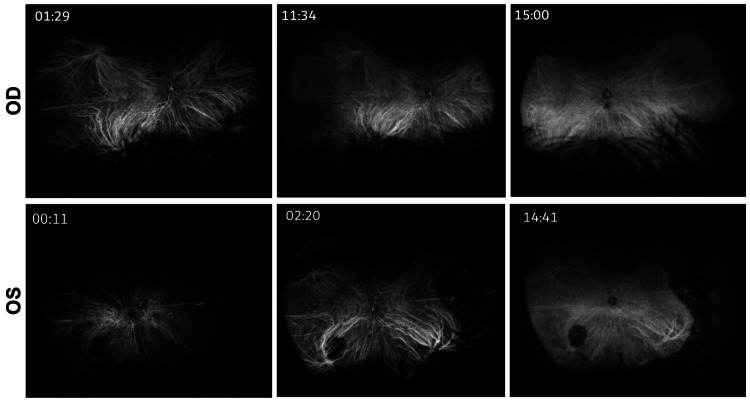
Serial indocyanine green angiography findings at follow-up (2024 visit) Indocyanine green angiography obtained in the early, mid, and late phases. Top row (OD): no major abnormality. Bottom row (OS): multiple hypocyanescent choroidal lesions/spots corresponding to the affected areas, with persistent abnormalities across sequential phases. These findings provided additional evidence of choroidal involvement and complemented the fundus, optical coherence tomography, fluorescein angiography, and ultrasonographic findings. OD, oculus dexter (right eye); OS, oculus sinister (left eye)

During follow-up, visual acuity in the left eye improved from 20/80 at presentation to 20/50 by six months and to 20/40 at the most recent evaluation in January 2024 (Figures 2E, 2F). The anterior chamber fibrinoid material resolved (Figure 6). Choroidal lesions partially regressed and then remained stable with residual pigmentary change. Optical coherence tomography showed resolution of subretinal fluid with mild residual choroidal thickening. B-scan ultrasonography showed no new mass lesion. During follow-up, indocyanine green angiography revealed normal choroidal vasculature in the right eye, whereas the left eye showed persistent hypocyanescent spots corresponding to the previous sites of choroidal infiltration (Figure 7). Surveillance computed tomography and positron emission tomography/computed tomography showed no systemic recurrence, and brain magnetic resonance imaging remained negative for the central nervous system disease. The chronological clinical course is summarized in Table 1.

**Table 1:** Chronological summary of the clinical course Abbreviations: ALK1, anaplastic lymphoma kinase 1; BCL1, B-cell lymphoma 1; BCL2, B-cell lymphoma 2; BCVA, best-corrected visual acuity; CD, cluster of differentiation; CNS, central nervous system; CT, computed tomography; DLBCL, diffuse large B-cell lymphoma; FA, fluorescein angiography; FISH, fluorescence in situ hybridization; HLA-A29, human leukocyte antigen A29; IOP, intraocular pressure; MRI, magnetic resonance imaging; MYD88, myeloid differentiation primary response 88; OCT, optical coherence tomography; OD, oculus dexter (right eye); OS, oculus sinister (left eye); PET/CT, positron emission tomography/computed tomography; mmHg, millimeters of mercury.

Time point	Clinical event	Key findings	Management/outcome
March, 2017	Right testicular diffuse large B-cell lymphoma diagnosed	Orchiectomy performed; reported immunophenotype included CD3-negative, CD5-positive, CD20-positive, BCL1-negative, BCL2-positive, ALK1-negative; FISH not available	Treated systemically outside the available ophthalmic record; achieved remission.
~August–December, 2021	Gradual onset of ocular symptoms	Painless progressive visual loss in the left eye for four months; no pain, redness, photophobia, floaters, or flashes; no fever, night sweats, weight loss, or neurologic symptoms	Referred for ophthalmic evaluation.
November, 2021	Preoperative ocular imaging	B-scan demonstrated diffuse choroidal thickening with no vitreous opacities at presentation	Supported suspicion for choroidal-predominant intraocular pathology.
December 2021 (initial ophthalmic examination)	Presentation to ophthalmology	BCVA 20/30 OD and 20/80 OS; IOP 13 mmHg OD, 20 mmHg OS; OS with inferior anterior chamber fibrinoid clumps/hemorrhage, yellow-white choroidal infiltrates, subretinal fluid, and mild disc swelling; OCT/FA/B-scan supported choroidal involvement; infectious evaluation negative; HLA-A29 positive	Differential included inflammatory and neoplastic masquerade syndromes; multimodal imaging and laboratory workup pursued.
December 2021	Diagnostic pars plana vitrectomy with vitreous biopsy	Retinal detachment identified intraoperatively and repaired with drainage retinotomy, scleral buckle, endolaser, and silicone oil injection; cultures negative; MYD88 L265P negative; cytology nondiagnostic; flow cytometry showed a lambda-restricted monoclonal B-cell population on a limited panel	Overall clinicopathologic picture favored secondary intraocular lymphoma with choroidal involvement.
February 2022	Systemic restaging Treatment initiated	Chest CT and PET/CT showed no active systemic lymphoma. Ocular relapse suspected in the absence of systemic/CNS disease.	Supported treatment planning for isolated ocular relapse. Ibrutinib 560 mg orally once daily started after multidisciplinary discussion.
~6 months after treatment initiation	Early follow-up	Visual acuity improved to 20/50 OS; anterior chamber fibrinoid material decreased; OCT showed improvement in subretinal fluid	Continued oral ibrutinib with ongoing surveillance.
November 2022	CNS surveillance	Brain MRI showed no central nervous system involvement	Continued systemic therapy and observation.
2023 surveillance	Ongoing systemic monitoring	Brain MRI, CT chest/abdomen/pelvis, and PET/CT remained without evidence of recurrent systemic lymphoma	No change in treatment strategy.
January 2024	Most recent ophthalmic follow-up	BCVA 20/30 OD, 20/40 OS; resolution of anterior chamber fibrinoid material; choroidal lesions partially regressed, then remained stable with residual pigmentary change; OCT showed resolution of subretinal fluid with mild residual choroidal thickening; no systemic recurrence	Durable disease control on oral ibrutinib over approximately 24 months.

## Discussion

This case illustrates late ocular relapse in a patient with prior testicular DLBCL, a lymphoma subtype well recognized for dissemination to immune-privileged sites [3-5]. The five-year interval between primary testicular disease and ocular presentation is clinically important because it shows that prolonged remission does not eliminate the risk of ocular relapse [4,5].

The presentation of unilateral yellow-white choroidal infiltrates, subretinal fluid, and anterior chamber fibrinoid material with little initial vitreous haze broadened the differential diagnosis to include posterior uveitis, Vogt-Koyanagi-Harada-like inflammatory disease, birdshot chorioretinopathy, infectious chorioretinitis, and other masquerade syndromes [6,8,9]. In this setting, the patient’s age, unilateral disease, prior systemic lymphoma, progressive, untreated course, and multimodal imaging findings all increased suspicion for malignancy rather than primary inflammation.

The diagnostic workup also highlights a common challenge in ocular lymphoma: routine cytology may be nondiagnostic because of low cellularity and the fragility of lymphoma cells [7]. In this patient, cytology did not show definite malignant cells, and the flow cytometric panel was limited. For that reason, we avoided claiming definitive histopathologic proof of recurrent DLBCL. Instead, the diagnosis relied on clinicopathologic correlation supported by a monoclonal vitreous population, typical choroidal infiltration on imaging, negative infectious workup, and the patient’s oncologic history. The negative MYD88 L265P result did not exclude lymphoma and was more compatible with secondary ocular involvement than with classic primary vitreoretinal lymphoma [7]. Regardless of the explanation, the vitreous flow cytometry supports a monoclonal B-cell process; however, in the absence of morphologic confirmation and given the immunophenotypic discordance with the primary tumor, it does not independently establish clonal relatedness to the prior testicular DLBCL. 

We cannot exclude some contribution from the anti-inflammatory properties of ibrutinib; however, the presence of a lambda-restricted B-cell population comprising 45.2% of CD45+ events, together with the ocular imaging findings, prior lymphoma history, and exclusion of major infectious etiologies, favors secondary intraocular lymphoma over a purely inflammatory process.

Treatment of secondary intraocular lymphoma with choroidal involvement is not standardized and should be individualized according to the extent of ocular, systemic, and central nervous system disease [8,9]. High-dose methotrexate-based regimens remain important, particularly when central nervous system involvement is present, but toxicity can be substantial in older adults. Ibrutinib was selected in this case because restaging did not show active systemic disease, the team wished to avoid more intensive chemotherapy, and emerging evidence suggests Bruton tyrosine kinase inhibition has activity in intraocular and central nervous system lymphoma. In the phase II iLOC study, ibrutinib 560 mg/day showed clinical activity in recurrent or refractory primary central nervous system lymphoma and primary vitreoretinal lymphoma, and long-term follow-up confirmed durable complete responses in a subset of patients [10,11]. A prospective phase II study of Bruton tyrosine kinase inhibitors in vitreoretinal lymphoma likewise reported high early disease-control rates with acceptable tolerability [12]. Although these data derive mainly from primary central nervous system and primary vitreoretinal lymphoma rather than secondary ocular relapse after testicular DLBCL, they provide a biologically plausible rationale for the favorable course observed here.

This case report has several important limitations. First, a choroidal biopsy was not performed. Although a vitreous biopsy was obtained, the choroidal infiltrates were the predominant abnormality, and direct choroidal tissue would have provided more definitive histopathologic confirmation. Second, the flow cytometry analysis was performed using a limited immunophenotypic panel, and the original flow cytometry plots were not available for re-review. In addition, cytologic preparations were acellular or paucicellular, and no morphologic correlate to the abnormal vitreous B-cell population was identified. Third, the CD5 immunophenotypic discordance between the primary testicular tumor (reported as CD5-positive) and the vitreous B-cell population (CD5-negative) limits our ability to establish definitive clonal relatedness. Without comparative molecular clonality testing between the primary and ocular specimens, the possibility of a second, unrelated B-cell neoplasm cannot be completely excluded. Fourth, although the vitreous flow cytometry findings support a monoclonal B-cell process, we cannot completely exclude the possibility that the clinical response to ibrutinib was influenced in part by its anti-inflammatory effects. Fifth, the original systemic chemotherapy regimen for the testicular DLBCL could not be confirmed from the available records, and fluorescence in situ hybridization for MYC, BCL2, and BCL6 rearrangements was not available for the primary tumor. Finally, this report describes a single patient, and the favorable outcome observed with ibrutinib may not be generalizable to other patients with suspected secondary intraocular lymphoma. Larger studies are needed to better define the diagnostic approach and optimal treatment for this rare condition.

## Conclusions

Secondary intraocular lymphoma with choroidal involvement may present years after treatment of testicular DLBCL and can mimic inflammatory chorioretinal disease. In patients with a prior history of lymphoma, unilateral atypical choroidal lesions with subretinal fluid should prompt early consideration of ocular relapse and careful long-term surveillance.

In this case, multimodal imaging, ocular fluid analysis, and systemic restaging were essential for reaching a clinicopathologic diagnosis favoring secondary intraocular lymphoma in the absence of definitive tissue confirmation. The detection of a lambda-restricted B-cell population by flow cytometry, comprising 45.2% of CD45+ events, provided important supportive evidence. Oral ibrutinib was associated with sustained clinical stability and visual improvement over 24 months, suggesting that BTK inhibition may be a reasonable individualized option for selected patients with apparently isolated ocular relapse who are not candidates for more intensive chemotherapy. Larger studies are needed to clarify the role of targeted therapy in secondary intraocular lymphoma.

## Appendices

Appendix A

**Table 2:** Ancillary molecular, immunogenetic, and systemic imaging findings relevant to diagnostic evaluation and staging Abbreviations: CNS, central nervous system; FDG, fluorodeoxyglucose; MRI, magnetic resonance imaging; NGS, next-generation sequencing; PET/CT, positron emission tomography/computed tomography; TI-RADS, Thyroid Imaging Reporting and Data System; TRB, T-cell receptor beta; TRG, T-cell receptor gamma.

Date	Study / test	Key reported findings	Relevance to ocular diagnosis / staging	Important limitation / interpretive note
11/29/2021	HLA typing (blood)	HLA-A29 positive; HLA-B27 negative; HLA-B51 negative	HLA-A29 positivity broadens the inflammatory differential, whereas negative HLA-B27 and HLA-B51 do not support acute anterior uveitis or Behçet-associated disease	HLA-A29 positivity is not diagnostic and cannot confirm or exclude lymphoma
11/29/2021	MRI brain with and without contrast	No intracranial metastatic disease; no suspicious orbital lesions; focal dural thickening along the left frontal lobe favored to represent calcified meningioma	Supports absence of overt CNS or orbital involvement at baseline systemic evaluation	Negative brain/orbital MRI does not establish the nature of the intraocular lesion
01/18/2022	MYD88 mutation analysis, non-blood ocular specimen	Negative for MYD88 p.Leu265Pro (c.794T>C) mutation	Does not provide molecular confirmation of lymphoma	Negative result does not exclude a lymphoid neoplasm; report states interpretation must be integrated with clinical and pathologic findings
01/18/2022	T-cell receptor gene rearrangement by NGS, non-blood ocular specimen	Negative for TRG/TRB rearrangement	Does not support a T-cell lymphoproliferative process	This is not a confirming test for secondary B-cell intraocular lymphoma
02/03/2022	PET/CT FDG skull to mid-thigh	No hypermetabolic abnormalities to suggest residual or recurrent lymphoma; no mass, fluid collection, inflammatory change, or pathologic adenopathy	Supports absence of active systemic recurrent lymphoma at restaging and favors an apparently isolated ocular process	Negative PET/CT does not exclude intraocular lymphoma
11/07/2022	MRI brain with and without contrast	Normal for age; no mass, mass effect, enhancing lesion, or restricted diffusion	Supports absence of radiologic CNS involvement on surveillance	Does not provide histopathologic confirmation of ocular disease
08/25/2023	MRI brain with and without contrast	Stable brain MRI without mass, hemorrhage, acute infarct, pathologic enhancement, or leptomeningeal enhancement; abnormal left globe signal/deformity unchanged	Supports ongoing absence of CNS progression during follow-up	Persistent abnormal globe appearance is nonspecific and requires ophthalmic correlation
11/19/2023	PET/CT FDG skull to mid-thigh	No evidence of residual or recurrent lymphoma; no hypermetabolic adenopathy; new hypermetabolic right thyroid nodule noted	Supports continued absence of systemic lymphoma recurrence during follow-up	Incidental thyroid lesion is outside the ocular diagnostic question and does not clarify ocular histopathology
12/06/2023	Thyroid ultrasound	2.0 × 1.3 × 1.9 cm complex vascular right thyroid nodule; ACR TI-RADS TR3 (mildly suspicious)	Documents workup of incidental PET-avid thyroid finding	Not relevant to confirmation of secondary intraocular lymphoma

Appendix B 

**Table 3:** Detailed vitreous cytology and flow cytometry findings from ocular specimens Abbreviations: 7-AAD, 7-aminoactinomycin D; SSC, side scatter.

Date	Accession No.	Specimen / component	Gross description / specimen adequacy	Cytology / morphologic findings	Flow cytometry findings	Integrated interpretation / diagnostic relevance
12/16/2021	SHF-21-03680	A. Pure vitreous, left eye	Approximately 1.5 mL colorless cloudy fluid received	Acellular specimen; no intact nucleated viable cells for evaluation	Not applicable	Nondiagnostic cytology because of absent cellular material
12/16/2021	SHF-21-03680	B. Vitreous washing, left eye	Approximately 19 mL colorless cloudy fluid received in syringe; cell block paucicellular	Acellular specimen; no morphologic correlate identified on prepared slides; five cytospin preparations acellular	Not applicable	Nondiagnostic morphologic assessment because of very low cellularity
12/16/2021	SHF-21-03680	C. Flow cytometric immunophenotyping, left eye vitreous (diluted and undiluted combined)	Specimen type: vitreous fluid; cell count 0.07 K/uL; viability by 7-AAD: 96% in lymphocyte gate and 50% in monocyte gate	No cells available for morphologic evaluation on cytospin	Limited B-cell panel assayed: CD45, CD19, CD20, CD22, CD5, CD10, CD38, mKappa, mLambda, 7-AAD. Abnormal population comprising 45.2% of CD45+ events, expressing lambda surface light chain, CD19, CD20, and CD22, without expression of CD5 or CD10. Blasts not visibly increased on CD45/SSC plot. Lymphocytes contained mostly atypical B cells with a minor population of CD5+, CD19-negative T cells; no increase in CD19-negative/CD5-negative lymphocytes	Supports a monoclonal B-cell population in the vitreous, but remains not independently diagnostic of secondary intraocular lymphoma because morphologic confirmation was absent and the report explicitly stated that the differential diagnosis included low-grade B-cell lymphoma or monoclonal B-cell lymphocytosis, with need for clinical and imaging correlation
01/18/2022	SHF-22-00155	Repeat vitreous washing, left eye	Approximately 10 mL diluted vitreous in syringe and approximately 0.5 mL undiluted yellow fluid received	Proteinaceous debris, scattered retinal epithelial cells, macrophages, melanophages, and occasional spindle cells in a background of vitreous material; no evidence of malignancy	Not reported for this specimen in the uploaded biopsy report	Repeat specimen remained negative for morphologic evidence of lymphoma, which lowers diagnostic certainty and reinforces the limited sensitivity of ocular fluid cytology in paucicellular specimens
